# Oligosaccharides Isolated from MGO™ Manuka Honey Inhibit the Adhesion of *Pseudomonas aeruginosa*, *Escherichia Coli* O157:H7 and *Staphylococcus Aureus* to Human HT-29 cells

**DOI:** 10.3390/foods8100446

**Published:** 2019-10-01

**Authors:** Jonathan A. Lane, Julie Calonne, Helen Slattery, Rita M. Hickey

**Affiliations:** 1 Teagasc Food Research Centre, Moorepark, Fermoy, Co. Cork P61 C996, Ireland; helen.slattery@teagasc.ie; 2 Department of Biological Sciences, Cork Institute of Technology, Cork T12 P928, Ireland; july.c@hotmail.fr

**Keywords:** Manuka honey, oligosaccharides, anti-adhesion, *Escherichia coli*, *Pseudomonas aeruginosa*, *Staphylococcus aureus*

## Abstract

Historically, honey is known for its anti-bacterial and anti-fungal activities and its use for treatment of wound infections. Although this practice has been in place for millennia, little information exists regarding which manuka honey components contribute to the protective nature of this product. Given that sugar accounts for over 80% of honey and up to 25% of this sugar is composed of oligosaccharides, we have investigated the anti-infective activity of manuka honey oligosaccharides against a range of pathogens. Initially, oligosaccharides were extracted from a commercially-available New Zealand manuka honey—MGO™ Manuka Honey (Manuka Health New Zealand Ltd.)—and characterized by High pH anion exchange chromatography coupled with pulsed amperiometric detection. The adhesion of specific pathogens to the human colonic adenocarcinoma cell line, HT-29, was then assessed in the presence and absence of these oligosaccharides. Manuka honey oligosaccharides significantly reduced the adhesion of *Escherichia coli* O157:H7 (by 40%), *Staphylococcus aureus* (by 30%), and *Pseudomonas aeruginosa* (by 52%) to HT-29 cells. This activity was then proven to be concentration dependent and independent of bacterial killing. This study identifies MGO™ Manuka Honey as a source of anti-infective oligosaccharides for applications in functional foods aimed at lowering the incidence of infectious diseases.

## 1. Introduction

For millennia honey has been used for medicinal purposes. The ancient Egyptians, Chinese, Assyrians, Greeks, and Romans often consumed honey for treatment of pain and acute fever [1]. Indeed, historians have discovered many references to the application of honey in wound treatment and in oral health in ancient civilizations. Although the benefits of honey have been known for some time it was not until the 20th century that scientific studies reported on its anti-bacterial and anti-fungal activity and its value in treating infected surgical wounds, burns, and decubitus ulcers [2,3,4,5]. These discoveries have led to the development of various products, such as honey-containing wound gel and toothpaste, which assist conventional medicines in the fight against bacterial infection. Recently, honey has attracted increased interest due to the emergence of multi-drug resistant ‘superbugs’ and the need for alternative therapies to fight against infectious disease. Encouraging such interest is the fact that bactericidal components of honey have been shown to eliminate chronic and/or drug resistant infection in vivo [6,7,8,9,10,11,12]. Furthermore, honey can inhibit quorum-sensing networks used by pathogenic bacteria which could potentially reduce infection and disrupt virulence without the development of resistance [13,14]. Taking such studies into consideration, honey is now being recognized as a potential source of therapeutic agents capable of preventing chronic infections. 

Honey is predominantly composed of water (17–20%) and sugar (~80%) but also contains proteins, enzymes, amino acids, organic acids, polyphenols, carotenoid-like substances, maillard reaction products, vitamins, and minerals [15,16]. Glucose (31%) and fructose (38%) are the most abundant sugars in honey, however, di-, tri-, and oligo- saccharides can also be found [17]. These more complex sugars are formed during the ripening stage in which enzymes and acids of honey are more productive [18]. Several excellent reviews have been dedicated to the characterization of honey composition and myriad of health benefits [19,20,21,22]. Often, the health promoting activity of honey is attributed to factors such as its low water activity, pH, and hydrogen peroxide and non-peroxide components [16]. However, research on honey has identified oligosaccharides as a potential bioactive ingredient. For example, Sanz et al. [23] demonstrated the prebiotic potential of honey oligosaccharides using an in vitro fermentation system. Similarly, Jan Mei et al. [24] have shown that two types of wild honey, Malaysian and Tualang, can support the growth of *Bifidobacterium longum*. These studies attributed this activity to the presence of fructooligosaccharides and the advantages of selectively stimulating the growth of these bacteria include the development of a more ‘balanced’ gut microbiota and increased resistance against pathogenic colonization [25,26,27]. This resistance occurs as commensal bacteria, such as bifidobacteria and lactobacilli, share mucosal carbohydrate binding specificities with enteric pathogens such as *Campylobacter jejuni*, *Helicobacter pylori*, *Salmonella enterica* and *Escherichia coli*. Therefore, occupancy of host cell surface receptors by commensal bacteria can lead to blocking invading pathogens thereby preventing the emergence of a diseased state. This anti-adhesive activity has also been demonstrated for other food sourced oligosaccharides as they mimic host cell surface receptors and block the initial attachment and/or compete with pre-existing attachments of microorganisms and toxins. To date, research in this area has mainly focused on the anti-adhesive activity of probiotics and bovine (BMO) and human milk oligosaccharides (HMO) [28,29,30,31]. In the current study, oligosaccharides from a commercially available New Zealand manuka honey, MGO™ Manuka Honey (Manuka Health New Zealand Ltd.), were isolated and the total oligosaccharide fraction was screened for anti-adhesive activity against *Escherichia coli* O157:H7, *Listeria monocytogenes*, *Cronobacter sakazakii*, *Salmonella enterica* serovar Typhimurium, and *Pseudomonas aeruginosa*. The main objective of this study was to determine the potential of using MGO™ Manuka Honey as a source of anti-infective oligosaccharides.

## 2. Materials and Methods

### 2.1. Extraction of Oligosaccharide From Manuka Honey

Methylglyoxal Manuka Honey produced by European honeybees (*Apis mellifera*) and made from the nectar of the native New Zealand manuka bush *Leptospermum scoparium* was supplied by Manuka New Zealand Health (grade MGO 100 or 100 mg/kg methylglycoxal, batch NO. 020710). This honey was tested and certified for MGO potency (by measuring the levels of the compound methylglyoxal), purity and quality. Chemical tests for 3-Phenyllactic acid, 2-Methoxyacetophenone, 2-Methoxybenzoic acid, and 4-Hydroxyphenyllactic acid are performed on all MGO^TM^ honey. In addition, DNA testing is performed on MGO^TM^ honey using a multiplex qPCR for the detection of *Leptospermum scoparium* DNA from pollen verifying its botanical origin. The isolation of oligosaccharides from manuka honey was carried out as per Sanz et al. [23]. Briefly, manuka honey (1 g) was dissolved in 40 mL of MillQ water and added to 250 mL of 10% (*v*/*v*) ethanol in water containing 6 g of activated charcoal Darco G-60, 100 mesh (Sigma-Aldrich^®^, Co. Wicklow, Ireland). This mixture was stirred for 30 min and then filtered under vacuum to remove the unbound monosaccharides. The oligosaccharides were recovered from the charcoal by mixing with 250 mL of 50% (*v*/*v*) ethanol. The mixture was stirred for 30 min and subsequently filtered under vacuum. Ethanol was then removed using a rotary evaporator and the sample was freeze dried. 

### 2.2. Analysis of Honey Oligosaccharides

#### 2.2.1. Oligosaccharide Standards

The oligosaccharide standards Kojibiose, Nigerose, Erlose, and D-Panose were purchased from Carbosynth Ltd. (Berkshire, UK). Maltose, maltriose, glucose and fructose were purchased from Sigma-Aldrich^®^. Of the eight sugars, two were mono-saccharides (glucose and fructose), three were disaccharides (kojibiose, nigerose and maltose) and three were tri-saccharides (erlose, panose, and maltriose). These specific standards were selected based on their abundance in honey [32]. 

#### 2.2.2. High Performance Anion-Exchange Chromatography with Pulsed Amperometric Detection (HPAEC-PAD)

HPAEC-PAD was used to determine the oligosaccharide composition of the freeze-dried honey oligosaccharide powder. Analyses were performed on a Dionex ICS-3000 Series system (Dionex Corporation, Sunnyvale, CA, USA) equipped with an electrochemical detector. Carbohydrate separation was carried out by a CarboPac PA 100 (250 × 4 mm) connected to a CarboPac PA 100 guard column (Dionex Corporation, Sunnyvale, CA). The elution was carried out with the following gradient: 100 mM NaOH (Eluent A) and 100 mM NaOH, 500 mM NaAc (Eluent B) (*t* = 0–3 min 95% eluent A; *t* = 3–13 min 88% eluent A; *t* = 13–30 min 50% eluent A; *t* = 30–45 min equilibrated at 95% eluent A). Commercially available oligosaccharides (described above) were used as external standards.

### 2.3. Inhibition Studies

#### 2.3.1. Bacterial Culture Conditions

The bacterial strains used in this study are listed in Table 1. All strains were grown under aerobic conditions overnight at 37 °C in their respective media (listed in Table 1). For inhibition studies the bacterial cells in the late exponential or early stationary phase were harvested from media, washed three times in phosphate buffer saline (PBS) and re-suspended in cell culture media to a concentration of 1 × 10^8^ CFU/mL. 

#### 2.3.2. Cell Culture Conditions

The human colonic adenocarcinoma cell line, HT-29, was purchased from the American Type Culture Collection. HT-29 cells were routinely grown in McCoy’s 5A modified medium (Sigma-Aldrich^®^) supplemented with 10% fetal bovine serum (FBS). All cells were routinely maintained in 75 cm^2^ tissue culture flasks and incubated at 37 °C in 5% (*v*/*v*) CO_2_ in a humidified atmosphere. Cells were passaged when the confluency of the flask was approximately 90%. For inhibition studies, HT-29 cells were seeded into 12 well PVDF plates (Corning, Deeside, UK) at a density of 1 × 10^5^ cells/well. Cells were allowed to grow for 48 h and the media was changed to McCoy’s 5A modified medium supplemented with 2% FBS at least 24 h prior to inhibition studies. 

#### 2.3.3. Anti-Infective Assay

Bacteria harvested from broth after overnight growth at 37 °C were washed and diluted in McCoy’s 5A medium (2% FBS) (1 × 10^8^ CFU/mL) supplemented with 5 mg/mL extracted manuka honey oligosaccharides (MHO). This concentration was selected based on physiological concentrations of oligosaccharides present in human milk [33]. This mixture was incubated at 37 °C (5% CO_2_) for 1 h and then used to infect HT-29 cells. Infections were performed at different incubation times based on previously reported data [34]. Non-adherent bacteria were removed by washing the cells six times with PBS after 1 h (*L. monocytogenes*, *C. sakazakii*, *S. aureus*, and *Pseudomonas aeruginosa*) or 2 h (*E. coli* and *S. enterica*) incubation in 5% CO_2_ at 37 °C. The cell associated bacteria were recovered by lysing the HT-29 cells with Triton X-100 (0.1% *v*/*v*) in PBS at 37 °C which selectively disrupts host membranes but does not affect the viability of the bacterial cells [35]. Serial dilutions of the cell lysates were plated onto agar plates and incubated at 37 °C for 12 h after which bacterial CFU were counted. To determine the effect of oligosaccharide concentration on *Pseudomonas aeruginosa*, *S. aureus*, and *E. coli* infection of the HT-29 cells, the assay was repeated using 5, 2.5, 1.25, and 0.625 mg/mL of extracted honey oligosaccharides.

### 2.4. Effect of Honey Oligosaccharides on Bacterial Growth

To determine the growth of each bacterial strain in the presence and absence of the oligosaccharides, the bacteria were grown in optimal growth media and colonization media (McCoy’s 5A media supplemented with 2% FBS) supplemented with MHO (5 mg/mL). Briefly, bacteria harvested from an overnight culture were used to inoculate (1%) the test media. The growth of the bacteria was monitored by making serial dilutions of the inoculated media (0, 3, 5, and 24 h), plating onto BHI agar and incubating the plates for 18 h under aerobic conditions after which bacterial CFU were counted.

### 2.5. Statistical Analysis

All inhibition studies were carried out on at least three separate occasions in triplicate. Results are presented as mean ± standard deviations of replicate experiments. Graphs were drawn using Microsoft Excel and the unpaired student Test was used to determine statistically significant results. *p* ≤ 0.05 was considered significant. 

## 3. Results

### 3.1. HPLC of Manuka Honey Oligosaccharides

**Figure 1:**
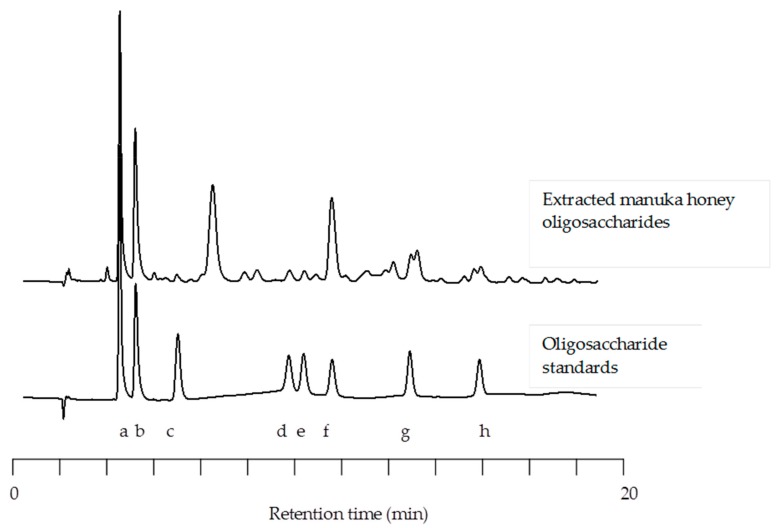
Comparative high performance anion-exchange chromatography with pulsed amperometric detection (HPAEC-PAD) chromatographs of manuka honey oligosaccharides with commercially available standards (Glucose (**a**), Fructose (**b**), Kojibiose (**c**), Nigerose (**d**), Maltose (**e**), Erlose (**f**), D-Panose (**g**), and Maltotriose (**h**)).

Extracted manuka honey oligosaccharides (MHO) were separated on a CarboPac PA100 column, detected using pulsed amperometric detection and quantified using external standards. HPAEC-PAD analysis revealed 28 peaks of interest. Due to the lack of commercially available standards many of these peaks could not be identified; however, those that could be identified and quantified included glucose, fructose, kojibiose, nigerose, maltose, erlose, D-panose and maltotriose. Although large amounts of glucose and fructose were removed during the extraction procedure; these simple sugars could be found in the final product (40 mg/g glucose and 48.5 mg/g fructose). The most abundant oligosaccharide structure quantified was erlose (179.5 mg/g) followed by panose (24.3 mg/g) and maltotriose (22.3 mg/g). Trace amounts of maltose (9.3 mg/g), nigerose (9.7 mg/g), and kojibiose (4.0 mg/g) were also identified (Figure 1).

### 3.2. Anti-Adhesive Activity of Manuka Honey Oligosaccharides

**Figure 2:**
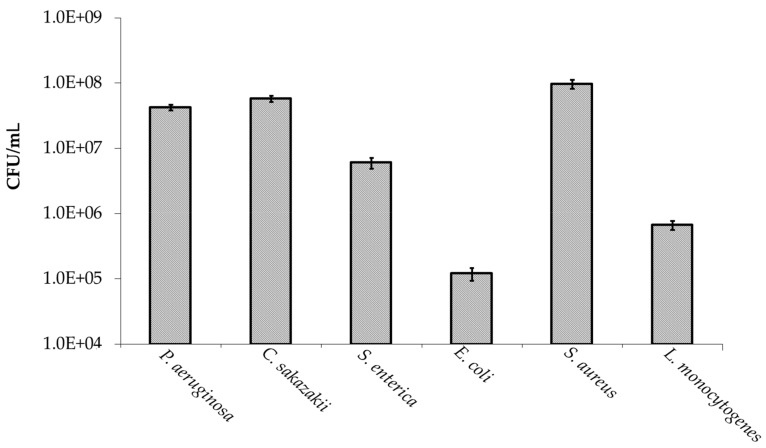
Adhesion of pathogenic bacteria to the human colonic adenocarcinoma cell line, HT-29.

Oligosaccharides extracted from manuka honey were screened for anti-adhesive activity against a range of pathogenic bacteria including *P. aeruginosa*, *C. sakazakii*, *S. enterica* serovar Typhimurium, non-toxigenic *E. coli* O157:H7, *S. aureus,* and *L. monocytogenes*. These oligosaccharides were shown not to be cytotoxic to the bacterial cells and did not affect the viability of the human cells as confirmed by simple trypan blue staining and viability studies on an xCELLigence system (Roche©). As illustrated in Figure 2, all pathogens screened had the capacity to adhere to the human colonic adenocarcinoma cell line, HT-29. High levels of adhesion were observed for *C. sakazakii* (10% of the initial inoculum), *P. aeruginosa* (54%) and *S. aureus* (64%) and moderate to low levels of adhesion were observed for *E. coli* O157:H7 (0.19%), *L. monocytogenes* (1.1%), and *S. enterica* serovar Typhimurium (2.2%). The adhesion of these bacteria to HT-29 cells was then assessed after pre-incubation with 5 mg/mL MHO. This initial oligosaccharide concentration was used as it was representative of the daily consumption (5–10 g/L) of human milk oligosaccharides by infants, which has been linked with preventing pathogen colonization within the gastrointestinal tract [33]. 

**Figure 3:**
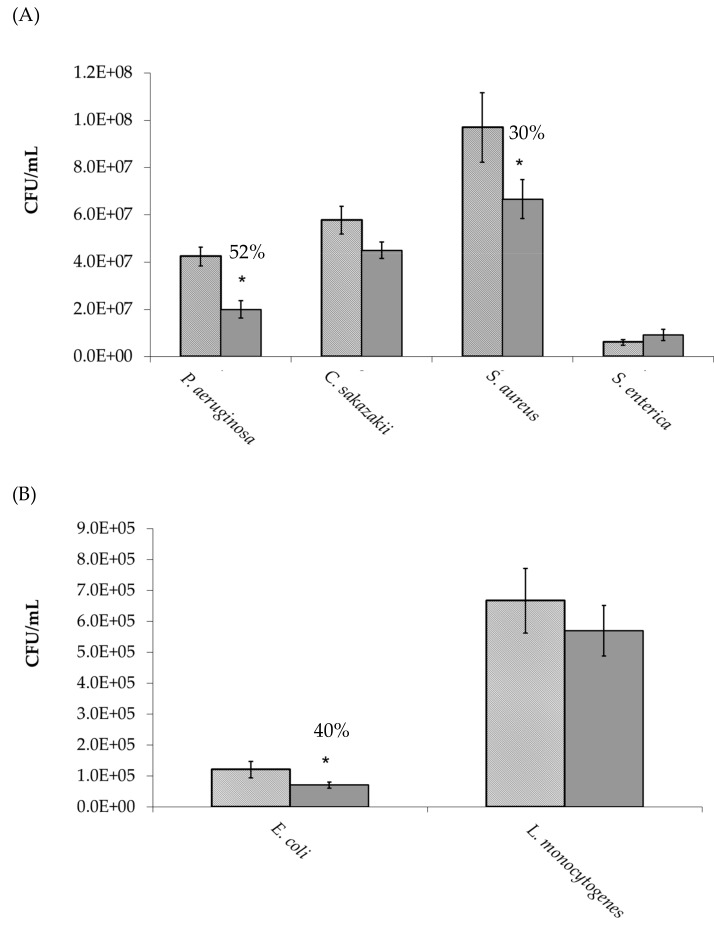
Adhesion of bacteria to HT-29 cells in the 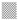 absence and 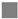 presence of manuka honey oligosaccharides. * *p* ≤ 0.05 was considered significant.

**Figure 4:**
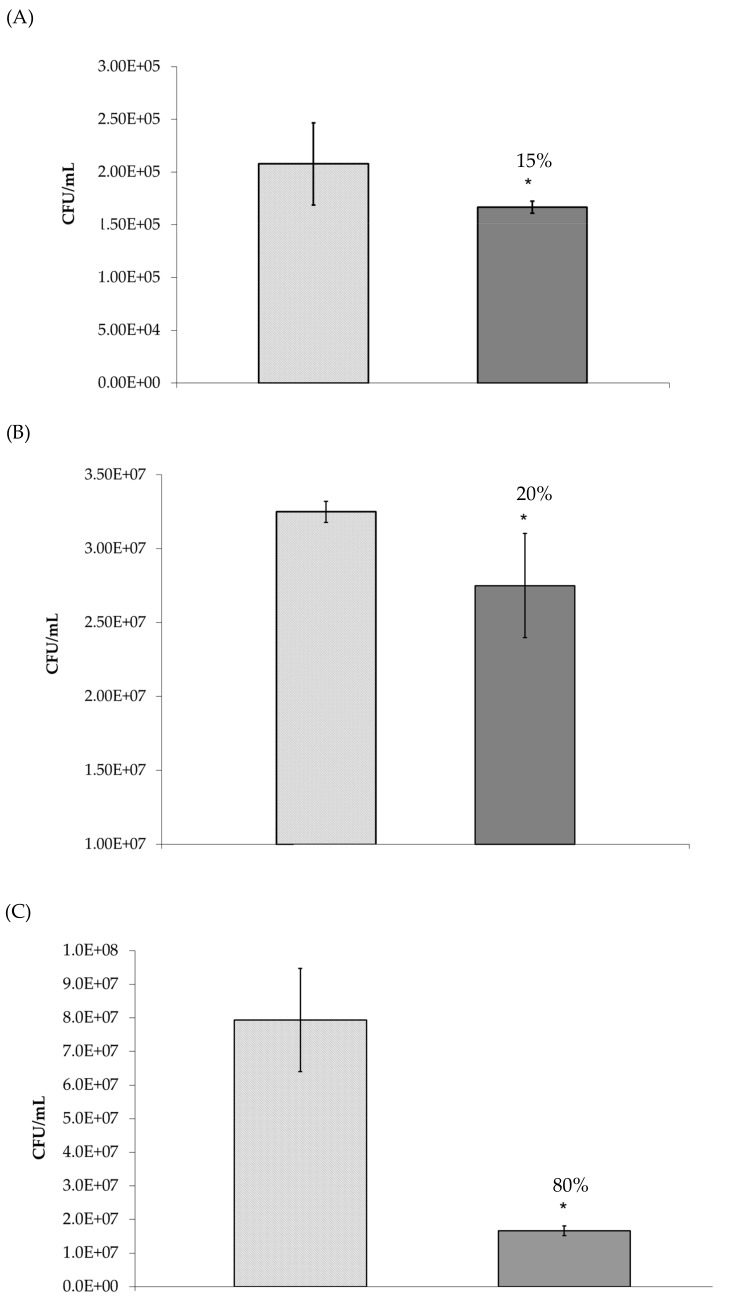
Adhesion of (**A**) *Pseudomonas aeruginosa*, (**B**) *Escherichia coli*, and (**C**) *Staphylococcus aureus* to HT-29 cells in the 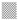 absence and 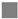 presence of manuka honey oligosaccharides where no pre-incubation step was performed. * *p* ≤ 0.05 was considered significant.

As observed in Figure 3A,B, *P. aeruginosa*, *S. aureus* and *E. coli* adherence to HT-29 cells was significantly (*p* ≤ 0.05) inhibited by 52%, 30%, and 40%, respectively, when compared to the control (no oligosaccharides). This anti-adhesive activity was then proven to be concentration dependent (Table 2). Indeed, *P. aeruginosa* adhesion to HT-29 cells was only reduced by MHO concentrations greater than 0.625 mg/mL and *S. aureus* and *E. coli* adhesion to HT-29 cells was only reduced by MHO concentrations greater than 1.25 mg/mL. As the pre-incubation of the bacteria with oligosaccharides prior to cell line infection may not be an accurate representation of an in-vivo situation; inhibition studies were also performed in the absence of this step (Figure 4). The oligosaccharides continued to demonstrate anti-adhesive activity under these conditions; however, the activity against *P. aeruginosa* and *E. coli* was significantly (*p* ≤ 0.05) reduced from 52% and 40% to 15% and 20%, respectively (Figure 4A,B). In contrast to this, the anti-adhesive activity of MHO against *S. aureus* dramatically increased to 80% under these conditions (Figure 4C). The MHO had no effect on the adhesion of the other pathogens examined (*L. monocytogenes*, *C. sakazakii,* and *S. enterica* serovar Typhimurium).

Growth analysis was also performed to ensure that the anti-adhesive activity observed for the extracted manuka honey oligosaccharide powder was not a consequence of bacterial cell death and to investigate the possibility that these oligosaccharides could increase the growth rate of the bacteria. When compared to the control (no oligosaccharides), no significant increase or decrease in bacterial cell numbers was observed for all bacterial strains tested at all time-points (3, 5, and 24 h) in the presence of the oligosaccharides (5 mg/mL). 

## 4. Discussion

To date, less than 30 oligosaccharide structures have been reported in honey [36]. The most common oligosaccharides identified in honey include panose, sucrose, maltose, kojibiose, isomaltose, erlose, trehalose, raffinose, and turanose [37,38,39,40]. These oligosaccharides are often found at varying concentrations with dependence on the source of the honey. For example, blossom honey (polyfloral) can be discriminated from honeydew honey (forest) as the latter contains a higher concentration of melezitose and raffinose [41]. Honeydew honeys also have lower contents of monosaccharides than blossom honeys [32]. Honeydew honeys have also been characterized by significantly higher mean values of trehalose and isomaltose, and lower values of glucose, sucrose and turanose, than blossom honeys. However, no significant differences in the mean amounts of fructose [42], maltose [42] and sucrose [43] were found while the mean value of total sugars in blossom honey was higher than that in honeydew honeys [42]. The sum of glucose plus fructose has also been used to distinguish between blossom honey and honeydew honey. Blossom honey must have a fructose plus glucose content ≥ 60% (*w*/*w*) while honeydew honey and blends of honeydew honey with blossom honeys must have a fructose plus glucose content ≥ 45% (*w*/*w*) (EU Directive 110/20010).

HPAEC-PAD is widely used to profile the oligosaccharide content in honey, of which there are more than 300 different varieties. For example, Ouchemoukh et al. [39] exploited this technology to profile Algerian honey oligosaccharides and Cotte et al. [44] and Morales et al. [38] demonstrated the use of this technology to detect honey adulterations. In our study, HPAEC-PAD was used to profile the total oligosaccharide fraction isolated from a commercially available New Zealand manuka honey (MGO™ Manuka Honey). Twenty-eight peaks of interest were identified with the most abundant oligosaccharides being maltotriose, panose, and erlose. These findings correlated well with previously published works such as that of Weston and Brocklebank [40] who reported on the presence of 20 oligosaccharides, including isomaltose, kojibiose, turanose, nigerose, and maltose, in New Zealand manuka honeys. Swallow and Low [45] also separated 20 structurally similar carbohydrates using HPAEC-PAD in four honeys of known botanical origin. The authors noted that although relative oligosaccharide concentration varied from one honey to the next, the overall oligosaccharide pattern did not differ significantly and therefore these oligosaccharide patterns could be used as a “fingerprint” for honey authenticity. The supplier of the honey used in this study (MGO™ Manuka Honey) adheres to international standards established by the Codex Alimentarius Commission. Therefore, the purity and quality of the honey is examined and C4 sugar analysis is performed to confirm no adulteration has taken place. For this reason, the overall oligosaccharide pattern among samples of this manuka honey may not vary greatly.

Overall, we concluded that this fraction was significantly depleted in monosaccharides and contained a complex and diverse range of oligosaccharides with potential biological activity. This fraction was subsequently screened for anti-adhesive activity against a range of pathogens and a significant reduction in the adhesion of *P. aeruginosa*, *E. coli* O157:H7 and *S. aureus* to human colonic epithelial cells, HT-29 cells, was observed. The fact that this fructose and neutral oligosaccharide enriched fraction demonstrated anti-adhesive activity against *P. aeruginosa* may not be surprising given that this bacterium has been shown to bind to both fucosylated and sialylated epitopes during colonization. Indeed, a variety of studies have shown that other honeys can interfere with the binding capacity of this nosocomial pathogen. For example, Lerrer et al. [46] reported that four commercial honeys (‘wild flower’, ‘eucalyptus’, and ‘field flower’ honeys) provided excellent hemagglutination-like protection against PA-IIL-mediated *P. aeruginosa* adhesion and attributed this activity to the interaction of *P. aeruginosa* PA-IIL, a fucose > fructose/mannose binding lectin, with fructose and fructooligosaccharides. Together these studies highlight honey as a food source capable reducing or preventing chronic colonization of *P. aeruginosa* in the gastrointestinal tract (GIT). Although in-vivo studies are required to substantiate this hypothesis, these are significant findings given the increased antibiotic resistance of this bacterium and the need for alternative therapeutic treatments to prevent *P. aeruginosa* infection. Similarly, *S. aureus* infection has become notoriously difficult to treat due to its resistance to antibiotics, such as methicillin [47]. *S. aureus* is a highly versatile pathogen found in the human pharynx, perineum, axilla, and on the skin (hands, chest and abdomen) and is mainly associated with wound infections, in which the bacterium gains access to the blood stream causing septic shock, and gastroenteritis. To date, relatively little has been reported on *S. aureus* interactions with food sourced oligosaccharides. Previously, we reported on a direct interaction between *S. aureus* and a dominant human milk oligosaccharide, 2’-fucosyllatose [48] and here, we report that manuka honey oligosaccharides can prevent *S. aureus* adhesion to colonic epithelial cells. Although further work in needed to investigate these interactions; these results suggest that carbohydrate-based compounds may have potential in preventing *S. aureus*-associated gastroenteritis once consumed. As previously discussed, manuka honey is commonly used to prevent infections, such as *S. aureus* infection, in minor wounds and burns. This was thought to be mainly due to its anti-bacterial and anti-inflammatory activity however, our results suggest that manuka honey oligosaccharides could also play an important anti-adhesive role. Indeed, these compounds could be binding directly to the bacterium and/or epithelial cell surface receptors which could neutralize the threat of bacterial colonization. 

*E. coli* O157:H7 is a highly virulent pathogen with an infectious dose as low as 5–50 cells and a major concern for the food industries such as the dairy industry [49]. The main source of this pathogen is bovine derived food products [50] and symptoms of infection include severe diarrhea, hemorrhagic colitis, and hemolytic-uremic syndrome. Various antibiotics and antibiotic combinations are often used to treat severe cases of *E. coli* O157:H7 infection and consequently, over the last 30 years, antibiotic resistant strains have emerged. Considering this, there is a need for alternative approaches to prevent and treat *E. coli* O157:H7 infections. Significantly, numerous *E. coli* strains have been shown to bind directly to food sourced glycans preventing their adhesion to target epithelial cell surface receptors. For example, Martin-Sosa et al. [51] reported on the ability of human milk oligosaccharides to prevent *E. coli* fimbriae-associated hemagglutination. Various groups have also demonstrated that bovine milk derived glycomacropeptide (GMP) reduces the adhesion of *E. coli* O157:H7 to human intestinal epithelial cells in-vitro [52,53]. Interestingly, this activity is mainly due to the presence of sialic acid at the terminal end of the *O*-linked glycans. Indeed, treatment of GMP with sialidase significantly reduced the binding of *E. coli* O157:H7 to GMP [53]. In our study, we demonstrate that manuka honey oligosaccharides can prevent the binding of *E. coli* O157:H7 to colonic intestinal epithelia in-vitro. Interestingly, honey does not contain sialylated oligosaccharides which would suggest that the activity of MHO is dissimilar to that of GMP. It should be noted that *E. coli* O157:H7 expresses multiple adhesins capable of binding both neutral and acidic oligosaccharides. Thus, neutralizing the threat of disease posed by this enteric pathogen through anti-adhesion therapy may require a complex mixture of oligosaccharides including neutral and acid oligosaccharides from various sources such as domestic animal milk and honey. 

During our initial screening studies, pathogenic bacteria were pre-incubated with honey oligosaccharides prior to cell line infection. As previously discussed, this is not an accurate representation of the potential use of these compounds. Thus, we examined the anti-infective activity of the oligosaccharides in the absence of this pre-incubation step. Positively, this did not eliminate the activity of the oligosaccharides however we did observe a slight reduction in activity against *Pseudomonas aeruginosa* and *E. coli* O157:H7. The reason for this could be that these pathogens bind directly to food sourced oligosaccharides, which prevents adhesion to epithelial cells [51,53] and under these conditions the concentration of oligosaccharides available for binding is reduced as these biomolecules also bind directly to epithelial cell surface receptors. Therefore, the availability of oligosaccharides for binding to *Pseudomonas aeruginosa* and *E. coli* O157:H7 in-vivo is an important factor to consider. Interestingly, the anti-adhesive activity of the MHO against *S. aureus* increased in the absence of a pre-incubation step. This suggests that epithelial-oligosaccharide interactions, such as occupancy of bacterial binding sites and/or changes in epithelial cell surface expression, can significantly contribute to preventing *S. aureus* adhesion to human epithelial cells. 

## 5. Conclusions

Overall, this study highlights MGO™ Manuka Honey as source of anti-infective oligosaccharides. Positively, these water-soluble biomolecules possess pH and thermal stability making them ideal for incorporation into dairy products, fruit juices, teas, snack bars, and other baked goods. Thus, the daily consumption of these compounds by individuals of all ages is possible and as demonstrated in this study could potentially reduce the risk of infection associated with multi-drug resistant bacteria such as *Pseudomonas aeruginosa*, *Escherichia coli* O157:H7, and *Staphylococcus aureus*. These biomolecules also exert a low selective pressure on invading pathogens as they are sourced from natural food products which would suggest that the risk of bacterial resistance is low. However, before an actual application can be considered, further questions must be addressed. The oligosaccharides are not 100% effective in preventing adhesion of the pathogens in this study. Would the concentration tested here in vitro be effective in vivo in humans? Also, would the cost of isolating oligosaccharides from manuka honey prohibit their use as food ingredients? The isolation method used in this study is not commercially viable and the amounts of honey required may not be available. A solution may be to identify the oligosaccharides in the honey responsible for bioactivity and synthesize these structures in a similar manner to human milk oligosaccharide manufacture (chemically, via fermentation or by enzymatic synthesis).

## Figures and Tables

**Table 1:** List of bacterial strains.

Pathogens	Growth Media	Strain information
*Staphylococcus aureus* ATCC 29213	BHI **	Human wound isolate
*Escherichia coli* DPC *P1432	BHI **	Non-toxigenic *E. coli* O157:H7 strain
*Salmonella enterica* serovar Typhimurium ATCC BAA-185	BHI **	Pig isolate
*Cronobacter sakazakii* NCTC 08155	BHI **	Infant formula isolate
*Listeria monocytogenes* NCTC 5348	BHI **	Isolated from mammal cerebrospinal fluid
*Pseudomonas aeruginosa* ATCC 33354	LB **	Serotype 6

* Dairy Products Research Centre, Teagasc, Moorepark, Fermoy, Co. Cork, Ireland. ** Brain heart infusion (BHI); Luria Broth (LB).

**Table 2:** Percentage Inhibition of adhesion.

	Concentration			
	5 mg/mL	2.5 mg/mL	1.25 mg/mL	0.625 mg/mL
*Pseudomonas aeruginosa*	46 ± 5.7	42 ± 10	34 ± 15	*-*
*Staphylococcus aureus*	51 ± 8.2	31 ± 10	-	*-*
*Escherichia coli O157:H7*	40 ± 13	20 ± 2.0	1 ± 9.0	*-*
